# Significance of PTEN Mutation in Cellular Process, Prognosis, and Drug Selection in Clear Cell Renal Cell Carcinoma

**DOI:** 10.3389/fonc.2019.00357

**Published:** 2019-05-08

**Authors:** Caibin Fan, Chunchun Zhao, Fei Wang, Shugen Li, Jianqing Wang

**Affiliations:** Department of Urology, The Affiliated Suzhou Hospital of Nanjing Medical University, Suzhou, China

**Keywords:** clear cell renal cell carcinoma, PTEN mutation, TCGA, RNA sequencing, bioinformatics analysis

## Abstract

It is well established that the PTEN (Phosphatase and Tensin Homolog) mutant is a frequently mutated gene found in clear cell renal cell carcinoma (ccRCC), making it a potential biomarker for individualized treatment opinions. Here, in the present study, we designed a method to evaluate the significance of the PTEN mutation in the prognosis and drug selection of ccRCC, determine the potential changing pathways and genes associated with the mechanisms. The most recent TCGA data shows that the PTEN mutation is found in 5% of ccRCC patients. In total, 2,569 genes were identified as DEGs. GO and KEGG analysis suggested that DEGs were significantly enriched in categories associated with cell division and multiple metabolic progressions. The top 10 genes, ranked by degree, were identified as hub genes from the protein–protein interaction network (PPI). What is more, patients with the PTEN mutation were associated with a worsened prognosis of ccRCC. Data from the GDSC database indicated that the selective AKT inhibitor, GSK690693, is a selective inhibitor for ccRCC with the PTEN mutation. Our findings have indicated that multiple genes and pathways may play a crucial role in PTEN mutation ccRCC, offering candidate targets and strategies for PTEN mutation ccRCC individualized treatment.

## Introduction

Renal cell carcinoma (RCC) or renal cancer accounts for 2–3% of all malignancies. Clear cell renal cell carcinoma (ccRCC) is a major subtype of RCC and is characterized by its resistance to chemotherapy, which can be identified by potential genetic mutations (1). Recent next-generation sequencing results revealed major frequent mutations including VHL, PBRM1, SETD2, BAP1, and KDM5C, thus dividing patients with different mutation types into different prognostic subtypes of ccRCC (2, 3). Patients with different mutations may be resistant to different treatments, thus making more individualized treatments for patients with different genetic conditions more important. Until now, the current targeted therapies for metastatic ccRCCs are mainly tyrosine kinase inhibitors that target angiogenesis rather than cancer cells themselves, with limited effects (4). Therefore, to determine potential biomarkers for disease progression and prognosis in ccRCC and potential corresponding targeted drugs is critical for individualized treatment.

PTEN (Phosphatase And Tensin Homolog), which contains a tensin like domain as well as a catalytic domain, similar to that of the dual specificity protein tyrosine phosphatases, serves as a multi-functional tumor suppressor that is mutated in a large number of cancers at high frequency, including ccRCC (5, 6). Alterations in PTEN expression may predispose RCC formation, which has potential prognostic and clinical significance (2, 7, 8). Functionally, PTEN negatively regulates intracellular levels of phosphatidylinositol-3,4,5-trisphosphate in cells and antagonizes the PI3K-AKT/PKB signaling pathway by dephosphorylating phosphoinositides, thereby modulating cell cycle progression and cell survival (9). Other studies also suggest that PTEN may function through AKT-independent mechanisms (10). Various signaling pathways and cell metabolic progressions might change in patients with the PTEN mutation compared to PTEN-wild type patients (9). Thus, a different PTEN status may affect cancer progression, disease prognosis, and treatment strategies, and result in natural resistance or sensitivity to treatment measures in some patients. Therefore, exploring the changes in various signaling pathways in patients with the PTEN mutation, and evaluating the significance in disease progression, will help us further understand the pathogenesis of the disease, providing more evidence for individualized treatment of ccRCC.

In this study, we analyzed an RNA sequencing (RNA-Seq) dataset of ccRCC and the GDSC database to identify the key pathways and genes associated with the PTEN mutation using bioinformatics analysis approaches, and evaluated the significance in drug selection, expecting to uncover the potential role of the PTEN mutation in serving as a prediction factor for prognosis and individualized treatment options.

## Materials and Methods

### RNA-Seq Data

An RNA-Seq dataset of renal clear cell carcinoma with corresponding clinical profiles was downloaded from The Cancer Genome Atlas (TCGA) database (https://portal.gdc.cancer.gov/). The corresponding information related to patients with the PTEN mutation was obtained from the cBioPortal for Cancer Genomics website (http://www.cbioportal.org/index.do)(11).

### Data Mining and Analysis of the GDSC Database

The Genomics of Drug Sensitivity in Cancer (GDSC) project is a collaboration between the Cancer Genome Project at the Wellcome Trust Sanger Institute (UK) and the Center for Molecular Therapeutics, Massachusetts General Hospital Cancer Center (USA), and is funded by the Wellcome Trust (12). To facilitate the visualization of the astronomical data matrix, the public online platform was developed with all data downloadable and plots reproducible (13). We first searched for compounds with significant selectivity for the PTEN mutation. We then tried to narrow the hits down, by checking the sensitivity only in ccRCC cells. The volcano plots, elastic nets, scatter plots, and the Mann-Whitney-Wilcoxon (MWW) tests were generated and computed via the GDSC online platform.

### Gene Set Enrichment Analysis (GSEA)

We analyzed GSEA v3.0 (http://software.broadinstitute.org/gsea/downloads.jsp) to dig out the differences in gene mRNA expression levels of biological functional annotation and pathways between PTEN mutation and wild-type patients, which helped us understand the effects of the PTEN mutation on various biological function gene sets, in renal clear cell carcinoma patients. The number of permutations was set at 5. Enrichment results satisfying a nominal *P*-value cutoff of 0.05 with a false discovery rate (FDR q-val) < 0.25 were considered statistically significant.

### Identification of Differentially Expressed Genes (DEGs)

The EdgeR, an R package for examining differential expression of RNA-Seq count data, was used according to the user's guide for screening differential expression of genes at gene levels between the PTEN mutation and wild-type renal clear cell carcinoma patients (14, 15). DEGs were identified with the following criterion: |fold change (FC)| ≥2; both the *P*-value and FDR < 0.05. The DEGs were used for further bioinformatics analysis.

### Functional Annotation and Pathway Enrichment Analysis of DEGs

Gene ontology analysis (GO) is a common useful method for annotating genes and gene products, to identify characteristic biological attributes for transcriptome data. Comprehensively, the mapping of user's genes to the relevant biological annotation in the database for annotation, visualization and integrated discovery (DAVID) website (https://david.ncifcrf.gov/) is an essential foundation for the success of any high-throughput gene functional analysis (16). GO-analysis and KEGG analysis was carried out using the database for annotation, visualization and integrated discovery (DAVID) website, while specifying a *P* < 0.05 for statistical significance.

### Integration of Protein–Protein Interaction (PPI) Network and Module Analysis

Search Tool for the Retrieval of Interacting Genes (STRING) database is an online tool that is employed to develop DEGs-encoded proteins and protein–protein interaction networks (PPI) (17). STRING (version 9.0) covers 5214,234 proteins from 1,133 organisms. Cytoscape software was utilized to construct protein interaction relationship networks and to analyze the interaction relationship of the candidate DEGs encoding proteins. Those with a combined score >0.4 were selected as significant. Then, the PPI network was used for module screening by Molecular Complex Detection (MCODE) (scores >3 and nodes >4) in Cytoscape, a bioinformatics integration platform (18). Furthermore, we also analyzed the KEGG pathway enrichment for DEGs in the top three modules, respectively.

### Statistical Analysis

The Student's *t*-test was used to compare the PTEN mRNA expression level between the PTEN mutation and wild-type renal clear cell carcinoma tissue. The Kaplan–Meier method with log-rank test was used to calculate the clinical outcome between different PTEN groups by Graphpad. FDR in edgeR and GSEA were adjusted for multiple testing with the Benjamini–Hochberg procedure to control FDR, respectively (19, 20). A value of *P* < 0.05 was considered statistically significant. All the statistical analyses were conducted with Graphpad and R 3.3.0.

## Results

### Data Information

**Figure 1:**
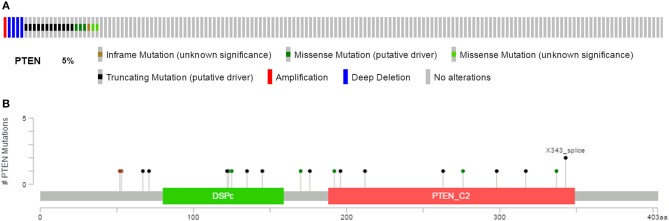
Mutation frequency **(A)** and types **(B)** of PTEN in ccRCC reproduced from the cancer Genome Atlas (TCGA) database.

We downloaded the information for 538 ccRCC patients and corresponding cancer tissue RNA-Seq datasets from the TCGA database, with complete follow-up profiles. There were 23 renal clear cell carcinoma patients (5%) with the PTEN mutation and the rest had the PTEN wild type (Figure 1A). Mutation types included amplification, truncating, deep deletion, inframe mutation and missense mutations spanning over entire gene (Figure 1B). These data were obtained from the cBioPortal for Cancer Genomics website.

### Clinical Impact of PTEN Mutation in ccRCC Progress and Prognosis

**Figure 2:**
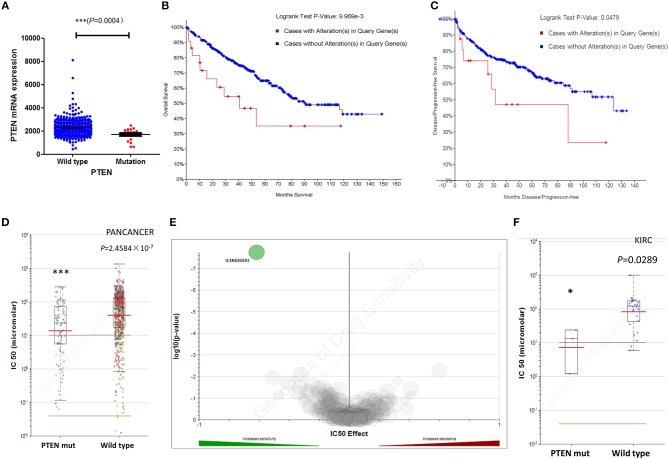
Mutations of PTEN are not associated with ccRCC prognosis and drug selection. **(A)** Correlation between the PTEN mutation and mRNA expression. **(B,C)** Kaplan–Meier survival and disease recurrence curves for renal clear cell carcinoma patients stratified by the PTEN mutation. **(D,E)** Scattered plot and volcano plot show that multiple cancer cell types with the PTEN mutation were significantly inhibited by GSK690693. **(F)** Reproduction of the GDSC database by excluding cancer of other types showed that RCC cells with the PTEN mutation was also significantly inhibited by GSK690693. *P < 0.05, ***P < 0.001.

We next investigated the influence of PTEN mutation on ccRCC progression and prognosis. The clinical characteristics of ccRCC Patients in both groups were list in Table 1. We first determined the PTEN mRNA expression level in the wild type and mutated group. Results showed that PTEN downregulated in mutated ccRCC patients' tumor tissue (Figure 2A). Analysis of the relationship between the PTEN status and disease prognosis showed that patients with the PTEN mutation had poorer prognosis on survival (Figure 2B) and disease recurrence (Figure 2C), which indicated that the PTEN mutation may contribute to ccRCC disease progression. Early intervention may be beneficial to patients with the PTEN mutation.

**Table 1:** Clinical characteristics of ccRCC patients and PTEN status in TCGA.

**Characteristics**	**PTEN status**	
	**Wild type**	**Mutated**
**Age, years**	60.6	60
Range	29–90	26–81
Gender		
Female	154	3
Male	276	15
**Tumor stage**		
T1	215	6
T2	54	3
T3	154	9
T4	7	0
**N stage**		
N0	196	9
N1	13	2
NX	221	7
**AJCC stage**		
Stage I	222	6
Stage II	45	3
Stage III	108	8
Stage IV	71	1
**Histologic grade**		
G1	9	0
G2	184	5
G3	169	7
G4	64	5
GX	3	1

### ccRCC Cells With PTEN Mutation Are Sensitive to GSK690693

In addition to the PTEN mutation on disease progression, we also investigated the role of the PTEN mutation in treatment of ccRCC patients. Current targeted therapy has a limited effect on metastatic ccRCC, and could easily induce drug resistance (21). With the scope of exploiting potent tumor inhibitors to help ccRCC individualized treatment, we studied the GDSC database to find whether PTEN mutated patients have potential selective compounds. Results showed that GSK690693 was a significant selectivity for the PTEN mutation in various cancer types (Figure 2D), making it a potential compound for patients with the PTEN mutation. We then studied the tissue specificity of GSK690693 and found that it exhibited sensitivity for renal cell carcinoma harboring the PTEN mutation in the GDSC database (Figures 2E,F). Altogether, we showed that GSK690693, a pan-Akt inhibitor targeting Akt1/2/3, conferred selective inhibition in ccRCC cells with the PTEN mutation, which made it a potential individualized compound for such ccRCC patients.

### GSEA

**Figure 3:**
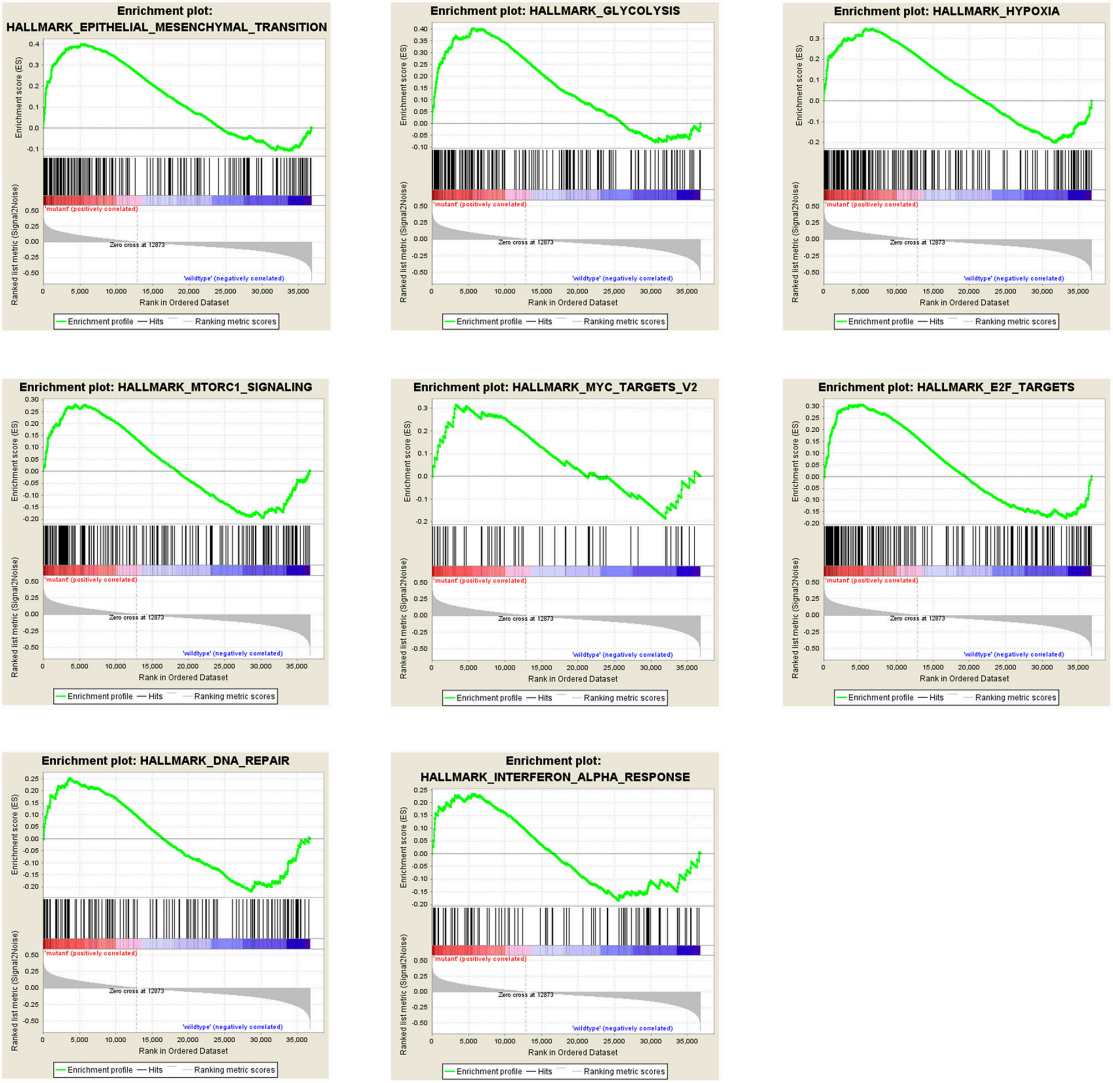
GSEA results of the PTEN mutation in ccRCC patients.

All results above shows that the PTEN mutation plays a critical role in ccRCC progression, prognosis and drug selection. To investigate the mechanism and to obtain some evidence, we first analyzed the effects of the PTEN mutation on cellular processes. At first, we analyzed various biological functional gene sets using the GSEA approach. As shown in Figure 3, results indicated that epithelial mesenchymal transition (EMT), glycolysis, hypoxia, mTORC1 signaling, MYC targets v2, E2F targets, DNA repair, and interferon alpha response were significantly enriched. These suggest that the PTEN mutation may promote ccRCC progression by influencing multiple pathways in cancer, migration, DNA repair and metabolism.

### Identification of DEGs

**Figure 4:**
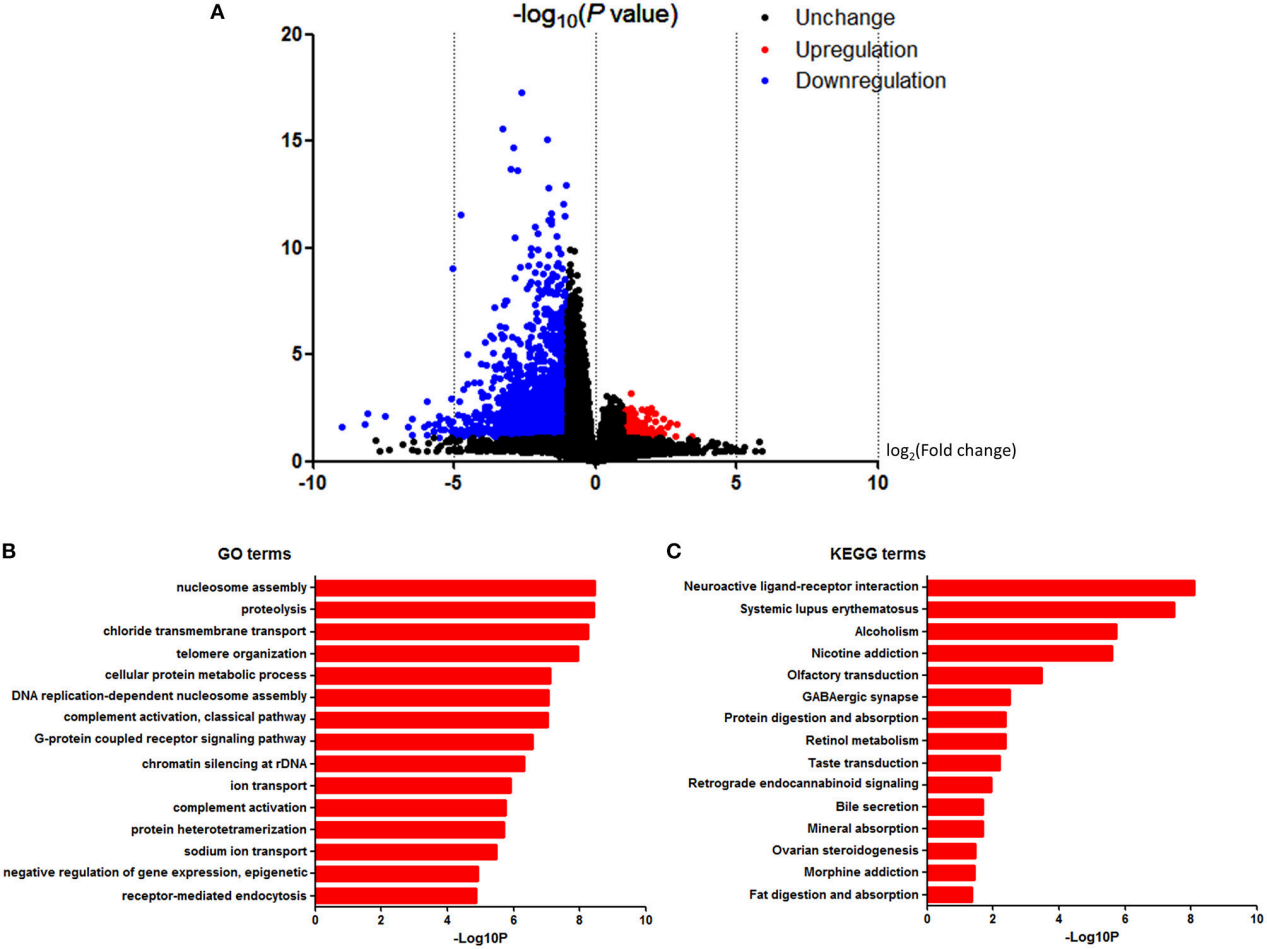
DAVID enrichment results of differentially expressed genes. **(A)** Volcano plot for differentially expressed genes. **(B)** The GO enrichment terms of differentially expressed genes. **(C)** The KEGG pathway analysis of differentially expressed genes.

To further investigate the pathways and genes implicated in the PTEN mutation, we identified the DEGs. RNA-Seq datasets from 23 PTEN mutation-bearing and other PTEN wild-type renal clear cell carcinoma patients were used for DEG screening. Based on the *in-silico* analysis, using |fold change (FC)| ≥ 2.0 and *P* < 0.05 criteria, a total of 2,569 genes were identified as DEGs. Among all genes mentioned above, we found that 207 were upregulated and 2,362 were downregulated. The volcano plot of the DEGs is shown in Figure 4A.

### GO and KEGG Analyses of DEGs

In order to analyze the DEGs at the functional level, we submitted all the 2,569 DEGs online for further GO and KEGG pathway analyses with DAVID, respectively. The GO analysis of DEGs (Figure 4B) suggested significant enrichment in the nucleosome assembly, proteolysis, chloride transmembrane transport, telomere organization, cellular protein metabolic process, DNA replication-dependent nucleosome assembly, complement activation, classical pathway, G-protein coupled receptor signaling pathway, chromatin silencing at rDNA, ion transport, complement activation, protein heterotetramerization, sodium ion transport, negative regulation of gene expression, epigenetic, and receptor-mediated endocytosis.

Furthermore, in the KEGG pathway analysis, DEGs were significantly enriched in the neuroactive ligand-receptor interaction, systemic lupus erythematosus, alcoholism, nicotine addiction, olfactory transduction, GABAergic synapse, protein digestion, and absorption, retinol metabolism, taste transduction, retrograde endocannabinoid signaling, bile secretion, mineral absorption, ovarian steroidogenesis, morphine addiction, and fat digestion and absorption (Figure 4C).

### Module Screening From the PPI Network

**Figure 5:**
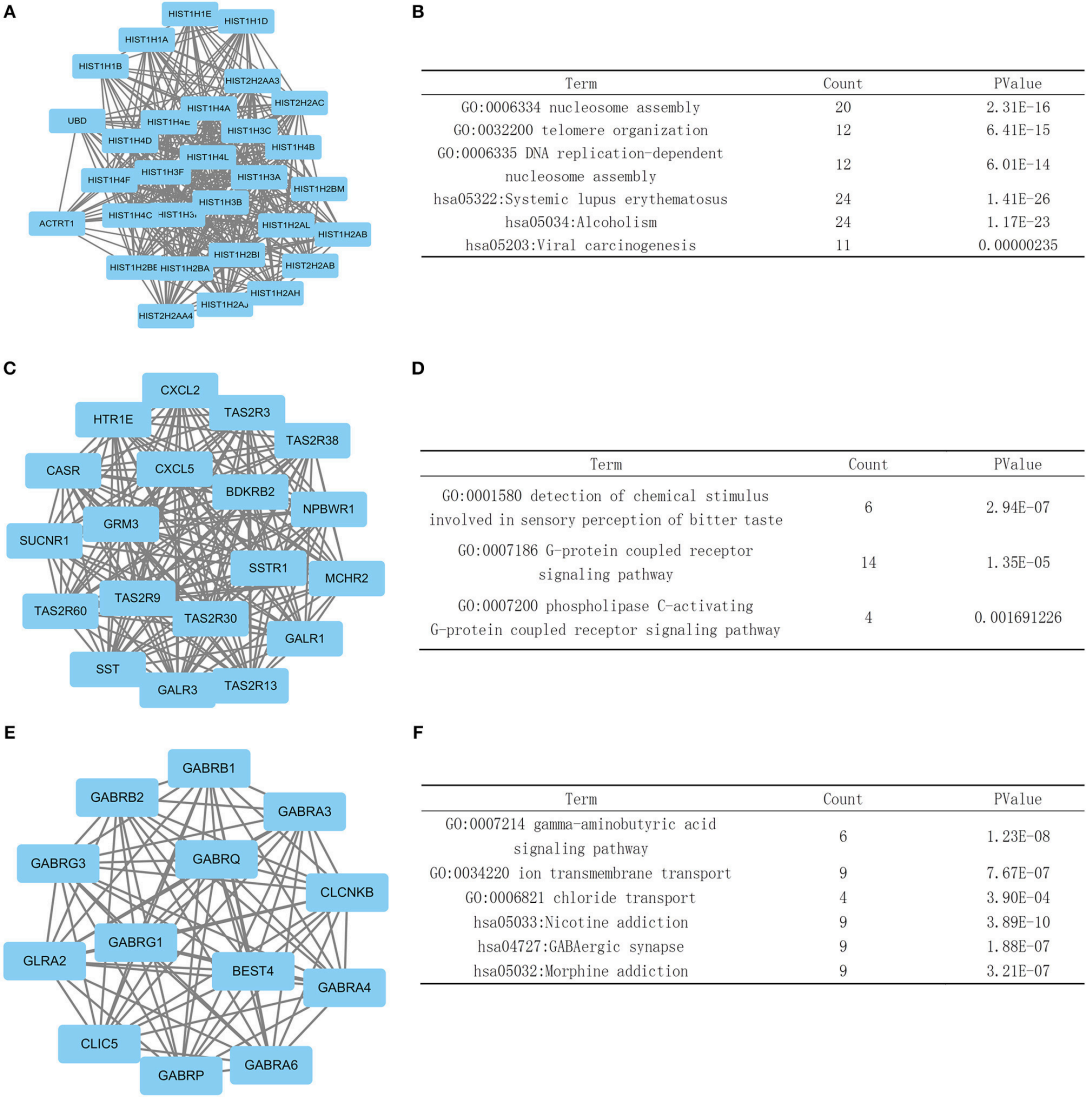
Top three modules from the PPI network. **(A,B)** PPI network and GO and KEGG analyses of module 1. **(C,D)** PPI network and GO and KEGG analyses of module 2. **(E,F)** PPI network and GO and KEGG analyses of module 3.

We then screened the information in the STRING database to investigate the interaction and hub genes of DEGs. The top 10 genes ranked by degree were identified as hub genes. These hub genes included PRKACG, EGF, SST, HIST2H2AC, HIST1H2BA, HIST2H2AA3, HIST1H2BM, HIST1H2BB, HIST1H4C, and HIST1H4A. PRKACG had the highest degree of nodes among the hub genes with 61. Modules of genes in the PPI network were identified by the MCODE plugin in Cytoscape. Then we did a GO and KEGG pathway enrichment analysis based on the top three significant modules. The top three significant modules were selected, and the functional annotation of the genes involved in the modules were analyzed (Figure 5). The enrichment analysis showed that the genes in module 1–3 were mainly associated with G protein- coupled receptors (GPCR) signaling pathway, gamma-aminobutyric acid signaling pathway, nucleosome assembly, telomere organization, and DNA replication.

## Discussion

PTEN, a tumor suppressor protein in many tumor types, acts as a dual-specificity protein phosphatase, dephosphorylating tyrosine-, serine-, and threonine-phosphorylated proteins, and also acts as a lipid phosphatase, removing the phosphate in the D3 position of the inositol ring from phosphatidylinositol 3,4,5-trisphosphate, phosphatidylinositol 3,4-diphosphate, phosphatidylinositol 3-phosphate, and inositol 1,3,4,5-tetrakisphosphate. The major function of PTEN relies on its phosphatase activity and subsequent antagonism of the PI3K/AKT pathway, while other studies also suggest that PTEN may function through AKT-independence (9). PTEN deletions and/or mutations are associated with a variety of human cancers, including renal cell carcinoma.

In this study, we intended to evaluate the clinical significance of the PTEN mutation in ccRCC progression, prognosis, and drug selection to promote individualized treatment. Related mechanisms were also analyzed. We found that about 5% of patients carried the PTEN mutation among 538 cases, including amplification, truncating, deep deletion, inframe mutation, and missense mutations spanning across the entire gene.

Clinical analysis showed that ccRCC patients with the PTEN mutation have a significantly poorer prognosis in survival and disease recurrence. All these results indicate the significance of the clinical practice of the PTEN mutation in ccRCC patients, consistent with some recent research (22, 23). The detection of tumor gene mutations has been applied to the clinic, which can help clinicians judge the prognosis of patients and effectively select better individualized treatment strategies. Our results provide evidence that ccRCC patients with the PTEN mutation are more prone to distant metastasis and that the prognosis of the disease is poorer, indicating that early intervention is required for such patients to obtain a longer survival period. CcRCC patients with the PTEN mutation may require more frequent follow-ups and more comprehensive examinations to detect early metastatic tumor tissues, or an earlier application of targeted drugs to cope with poor disease prognosis. What is more, data from the GDSC showed preliminary evidence that GSK690693 exhibited sensitivity for renal cell carcinoma harboring the PTEN mutation, which provides more evidence for the application of specific anti-tumor drugs to such patients and provides a basis for further research. Maybe in the future, this compound could be used in targeted drugs for ccRCC patients with the PTEN mutation for individualized treatment.

As for the mechanisms, we analyzed the RNA-Seq dataset of ccRCC downloaded from TCGA to identify the key pathways and genes associated with the PTEN mutation, using bioinformatics analysis approaches. GSEA analysis in the present study suggests that the PTEN mutation is significantly associated with multiple cancer related pathways, epithelial mesenchymal transition (EMT), DNA repair, and metabolism, such as glycolysis, hypoxia, mTORC1 signaling, MYC targets v2, E2F targets, DNA repair, and interferon alpha response. Among the processes mentioned above, EMT is a complex biological process in which genetic, and epigenetic events lead to epithelial cells acquiring a mesenchymal gene activity signature and phenotype. This process occurs in normal morphogenetic processes and in pathological situations, which is also an important process thought to contribute to cancer cell migration and metastasis (24, 25). Our results indicated that the tumors of patients with the PTEN mutation may be more prone to distant metastasis, supporting its value in clinical applications. As PTEN is one of the key inhibitors of the PI3K-AKT signaling pathway, the PTEN mutation definitely affects the activity of the mTORC1 signaling pathway.

We then identified the DEGs in both groups and carried out the GO term and KEGG analysis. Results showed that about 2,569 genes were DEGs with a different mutation status of PTEN. An enrichment analysis indicated that DEGs in the PTEN mutation ccRCC patients were related to biological processes of muscle contraction, G-protein coupled receptor signaling pathway, metabolism, DNA replication, and nucleosome assembly, which implies that patients with the PTEN mutation might have more vigorous cell energy metabolism and cell growth. Previous research has confirmed that adaptations across multiple metabolic processes are necessary to satisfy the energy required for an increased rate of proliferation in the malignant transformation progression. Dysregulation of the cell metabolism, such as increased lipid accumulation, has been a hallmark of the malignant phenotype. Changes in the levels of a variety of lipid metabolic enzymes has been documented in renal clear cell carcinoma, which also provides various treatment targets (26). Therefore, the analysis above suggests the function of the PTEN mutation in cellular progression and provides insight into the promising therapeutic target in patients with the PTEN mutation for individualized treatment opinions.

In the PPI network analysis, we identified hub genes which consisted of the 10 DEGs with the highest degree of interaction. The PRKACG gene, which encodes Protein Kinase CAMP-Activated Catalytic Subunit Gamma, functions as the gamma form of the Cyclic AMP-dependent protein kinase (PKA) catalytic subunit. GO annotations related to this gene include transferase activity, transferring phosphorus-containing groups and protein tyrosine kinase activity. The KEGG and GO analysis of the top three significant modules in the PPI network showed enrichment in DNA replication, nucleosome assembly, G protein- coupled receptors (GPCR) signaling pathway and telomere metabolism, indicating that tumors with the PTEN mutation tend to be more active in cell division and cell signaling transport.

Our study contained one limitation. In our study, we could only investigate if the PTEN mutation in ccRCC influences disease progression, prognosis, and drug selective. The mechanism and validation of the PTEN mutation in ccRCC still needs to be further researched in clinical and molecular biology experiments.

In conclusion, this study determined the main pathways and genes associated with the PTEN mutation in ccRCC, which may facilitate the development of the PTEN mutation to improve ccRCC risk prediction through the development of therapeutic strategies against such special subtypes of ccRCC.

## Data Availability

Publicly available datasets were analyzed in this study. This data can be found here: https://portal.gdc.cancer.gov/.

## Author Contributions

JW conception and design, obtained funding, and drafted the manuscript. CF and CZ acquired the data and drafted the manuscript. CF critically revised the manuscript. FW and SL statistical analysis and technical support.

### Conflict of Interest Statement

The authors declare that the research was conducted in the absence of any commercial or financial relationships that could be construed as a potential conflict of interest.
